# Behavioral factors to modulate immunotherapy efficacy in cancer

**DOI:** 10.3389/fimmu.2022.1066359

**Published:** 2022-12-16

**Authors:** C. Jongerius, L. Vermeulen, M. van Egmond, A. W. M. Evers, L. M. Buffart, K. J. Lenos

**Affiliations:** ^1^Laboratory for Experimental Oncology and Radiobiology, Center for Experimental and Molecular Medicine, Amsterdam University Medical Centers location University of Amsterdam, Amsterdam, Netherlands; ^2^Cancer Center Amsterdam, Cancer Biology and Immunology, Amsterdam, Netherlands; ^3^Oncode Institute, Amsterdam, Netherlands; ^4^Department of Molecular Cell Biology & Immunology, Amsterdam UMC, Location VU University, Amsterdam, Netherlands; ^5^Department of Surgery, Amsterdam UMC, Location VU University, Amsterdam, Netherlands; ^6^Department of Health, Medical and Neuropsychology, Leiden University, Leiden, Netherlands; ^7^Department of Physiology, Radboudumc, Nijmegen, Netherlands

**Keywords:** behavioral medicine, cancer, immune checkpoint inhibition, exercise, stress, classical pharmacological conditioning

## Abstract

Immune checkpoint inhibitors, including anti-PD-1 and anti-CTLA-4 therapies, are used to (re)activate the immune system to treat cancer. Despite promising results, a large group of patients does not respond to checkpoint inhibition. In the vulnerability-stress model of behavioral medicine, behavioral factors, such as stress, exercise and classical pharmacological conditioning, predict cancer incidence, recurrence and the efficacy of conventional cancer treatments. Given the important role of the immune system in these processes, certain behavior may be promising to complement immune checkpoint inhibition therapy. Here, we discuss the preliminary evidence and suitability of three behavioral mechanisms, i.e. stress modulation, exercise and classical pharmacological conditioning for the benefit of immunotherapy. It is crucial to study the potential beneficial effects of behavioral strategies that support immunotherapeutic anti-tumor effects with rigorous experimental evidence, to exploit behavioral mechanisms in improving checkpoint inhibition efficacy.

## Introduction

Immune responses are the collective of biological processes aiming to protect an organism from pathogens, like bacteria, viruses and parasites (1). In addition, the immune system plays a pivotal role in limiting cancer formation through dedicated immunosurveillance mechanisms (2). Recent developments in immunotherapy have caused a revolution in the treatment of a number of malignancies, often drastically improving disease outcome (3). In particular, immune checkpoint inhibition (ICI) can be applied to target key suppressors of the immune system in order to treat cancer (4). By blocking the interaction between tumor cells and immune checkpoints on T cells, such as CTLA-4 or PD-1/PDL1, the break on T cell inhibition is released, enabling activation, proliferation and the release of cytotoxins such as perforin and granzymes that eventually lead to apoptosis of tumor cells (5–7). Despite promising results of ICI, immunotherapy is currently applicable to only a small proportion of cancers, of which only a limited number of patients respond (8–10). In addition, ICI therapy has several severe side effects associated to autoimmunity (11). Therefore, patients and healthcare at large would benefit from strategies to improve the efficacy of these treatments (3, 12–14).

**Figure 1:**
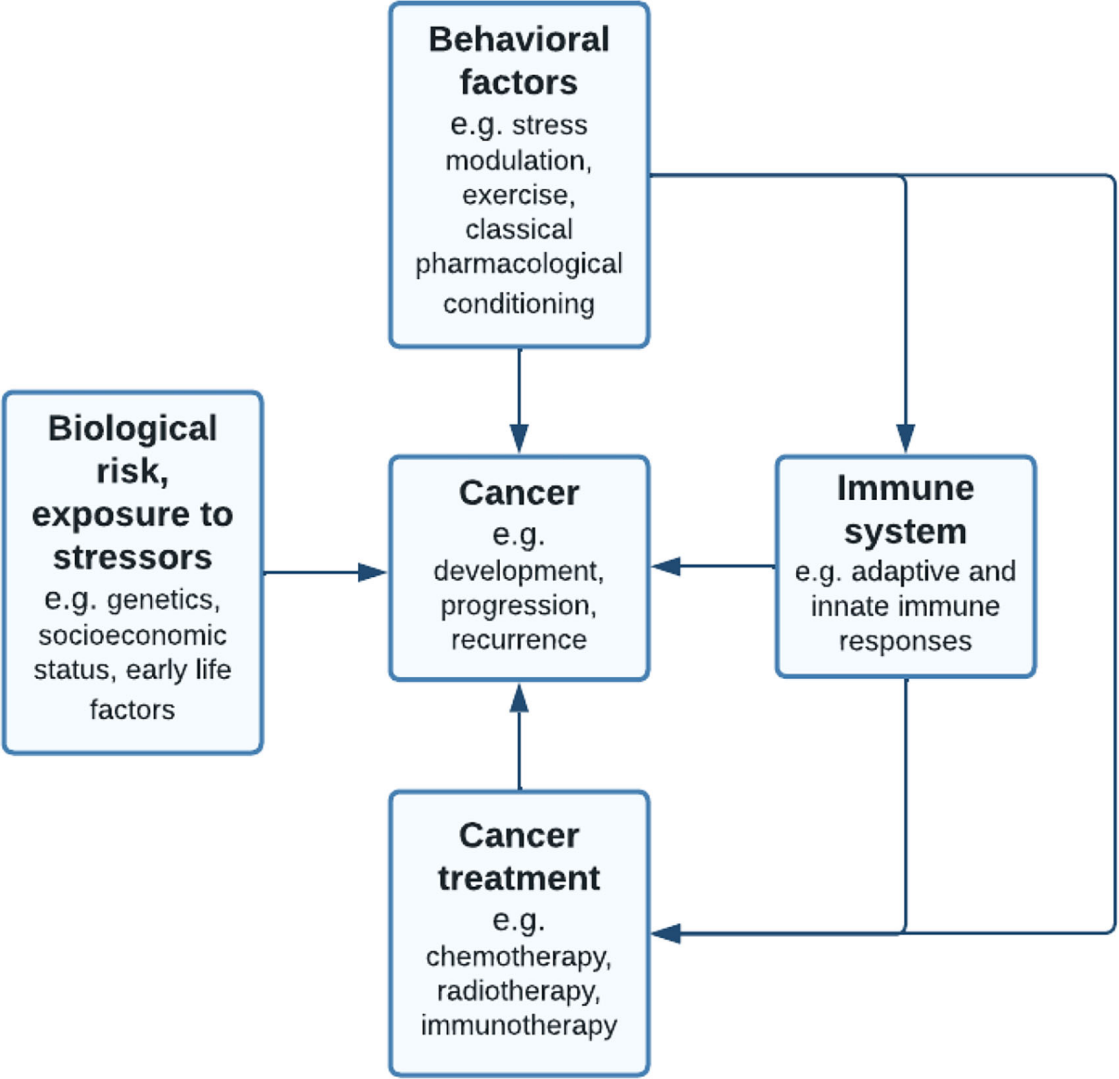
Adapted vulnerability-stress model (36): pathways linking behavioral factors to cancer. The behavioral medicine (37) model suggests that while individuals inherit biological risks such as genetic predisposition to cancer, this vulnerability requires interaction with stressors such as chronic traumatic events. Next to this, lifestyle behavior or behavioral interventions may influence cancer development, progression or recurrence, mediated by the immune system. Behavioral factors such as stress modulation, exercise and classical pharmacological conditioning are suggested to influence both conventional cancer treatments, such as chemotherapy, as well as immune checkpoint inhibition therapies.

Behavior, and consequently behavioral interventions, have been shown to broadly affect the immune system (15–26), and as such may help to improve therapeutic efficacy. Previous studies have shown that behavioral therapies improved quality of life and energy levels in patients receiving chemotherapy, radiotherapy or hormonal therapy (27–33). Also, preclinical studies have shown effects of behavioral therapies on clinical outcomes such as tumor growth, which could be partially mediated by the immune system (34, 35). Hence, we argue that the application of behavioral therapeutic approaches is especially relevant for immunotherapy. Nonetheless, research on behavioral interventions in relation to immunotherapy is scarce, as opposed to more conventional cancer therapies. While many pre-clinical behavioral interventions appear to benefit anti-cancer treatment effectivity, only few directly influence the immune system and therefore may serve as complementary to checkpoint inhibition treatment. Based on the vulnerability-stress model (36), we propose three behavioral applications in immune checkpoint therapy: management of the stress response, exercise, and classical pharmacological conditioning (**Figure 1**). Modulation of these behaviors can directly impact cancer development, progression or survival.

Here, we will first review the behavioral factors affecting immune responses during cancer progression and treatment. Next, we discuss the potential benefits of behavioral interventions to support checkpoint inhibitor therapies.

## Immune responses can incite or restrain cancer

The human immune system, often described as innate and adaptive immune responses (1, 38), is essential for the host’s survival by offering protection against pathogens through, for instance, cytokines, lymphocytes and antibodies. Innate immune responses recognize abnormal cell surface molecules, applicable to a broad group of pathogens, like viruses and bacteria, but, importantly, also tumor cells. The main cellular actors of the innate immune response, such as natural killer (NK) cells, and phagocytes, do not require a previous encounter to elicit responses. In contrast, adaptive immunity requires an initial encounter with an agent to mount an enhanced counterattack upon future encounters, providing a long-term immunological memory of specific pathogens. Adaptive immune responses consist of specialized lymphocytes, like T-lymphocytes (T cells). T cells, subdivided into the CD4^+^ T (helper) cells and CD8^+^ T (killer) cells, recognize peptide antigens that are presented on the cell surface *via* MHC molecules (3). T helper or killer cells respectively produce immune modulating cytokines or directly kill pathogenic cells by secreting perforin and granzymes (6). Perforin translocates to the target cell and binds to its cell membrane to cause pore formation (39). The pores allow diffusion of the granzymes into the target cell, activating cell death (39), and thereby complementing the innate immune system in clearing infected or tumor cells.

As a result of ongoing immunosurveillance the immune system can influence tumor onset, growth and therapy (2, 40). Paradoxically, immune responses may unintendedly shift from being tumor suppressive to supportive, for instance through inflammation. Acute and chronic inflammation both have distinct cellular profiles (41). Acute inflammation is characterized by a high presence of neutrophils, whereas chronic inflammation is featured by the presence of macrophages and lymphocytes (42). Both types of inflammation display inappropriate (dis)engagement of the immune system resulting in tissue remodeling and destruction, and even DNA alterations due to oxidative stress (43). As such, inflammation may also become uncontrolled, predisposing to tumorigenesis (44). Several cancer types, such as colorectal-, liver-, stomach- and bladder cancer may arise from sites of infection or chronic inflammation (45). In these tumors, albeit not exclusively, cancer cells engage with immune cells into an inflammatory tumor microenvironment, a prerequisite for most tumors (46). In inflammatory bowel disease, cancers are found predominantly at the sites of inflammation and chronic intake of anti-inflammatory medications has been shown to decrease the incidence of these cancers (47, 48), by putting a halt to the continuous recruitment of inflammatory cells that destroy the homeostasis of local tissues. During inflammation, the immune system releases reactive oxygen and nitrogen species (ROS and RNS), thereby causing DNA damage in proliferating cells (49). Oxidation is the most abundant type of DNA damage and is also able to inactivate DNA in a non-specific way, leading to accumulation of DNA lesions, genomic instability and cancer (50). An inflammatory microenvironment is not only essential to tumor onset, but also to tumor progression by sustaining tumor cell proliferation. This can be exerted by for instance tumor-associated macrophages with an M2-like profile (51), releasing angiogenic factors enhancing vascularization and thus promoting tumor growth (52). Subsequently, inflammation can also play a major role in the prognosis and treatment of cancer. For example, cancer therapy can trigger inflammatory responses by causing trauma and tissue injury, thereby stimulating tumor re-emergence and resistance to therapy (53). Hence, the use of anti-inflammatory agents, such as non-steroidal anti-inflammatory drugs, can be beneficial for the prognosis of patients (54, 55).

## Behavior modulates immune responses

Behavior and the immune system are interrelated. Exemplary is the behavioral immune system, which is the psychological mechanism that allow individuals to detect parasites or pathogens in their environment and avoid contact with the objects or individuals carrying them (56, 57). The behavioral immune system aims to avert infections through preventive behavior; therefore, behavior directly influences the immune system. Next to this, the immune system is responsive to behavioral factors such as stressors, exercise, and classical pharmacological conditioning. Those behavioral factors may modulate immune responses singularly as delineated below, however, they may also influence each other, e.g. exercise can reduce psychosocial stress (58).

Both physiological stress and psychosocial stress, i.e. exposure to a physical or social stressor like pain or social exclusion respectively, evoke a physiological stress response (59). Being confronted with stressors modulates the immune system by triggering the fight-or-flight response, which is a physiological reaction to a perceived harmful event (60). The physiological stress response depends on several factors, such as the stressor itself (e.g. duration), the host (e.g. age) and external factors (e.g. the environment) (61). Stressor perception induce the secretion of stress-related molecules, such as catecholamines and cortisol *via* the sympathetic nervous system or the hypothalamic-pituitary-adrenocortical (HPA) axis. Sympathetic fibers descend from the brain into lymphoid tissues, for example the thymus and the spleen, which release different substances that can bind to white blood cells, e.g. (nor)epinephrine (62). The HPA axis releases adrenal hormones that bind to white blood cells, regulating their distribution and function (62). In addition, managing stressful events may be demanding for individuals, leading them to engage in maladaptive behaviors as alcohol abuse or changes in sleeping patterns, which can modify immune system processes. Over 300 studies have investigated the different psychological challenges capable of modifying features of the immune responses, illustrating that the more a stressor becomes chronic, the more the immune system is compromised (62).

Analogously, a bout of exercise may lead to mobilization of different immune cells, including leukocytes, increased T cell activity and increased immune activity in general (63–65). Exercise is defined as physical activity that is planned, structured, repetitive and purposeful to improve or maintain physical fitness or health (66). It is generally accepted that prolonged exhaustive exercise training can depress immunity, while regular moderate intensity exercise is beneficial (67). The latter is illustrated by for instance decreased biomarkers of inflammation (for example c-reactive protein) in physically active as opposed to sedentary individuals (68).

Classical pharmacological conditioning is the third example of a behavioral mechanism that has been shown to influence the immune system. Classical conditioning is a learning process in which an initially neutral stimulus elicits a learned physiological response through repeated pairing of the stimulus and the physiological response. Ivan Pavlov first discovered these learned reflexes in 1927 (69) by training dogs to salivate at the presentation of a conditioned stimulus: the sound of a bell. Conditioning or learning is relevant for any human behavior and is therefore applied broadly, for example in psychoanalysis focused on social behavior. Here, we refer to the pharmacological form of classical conditioning, which is an instrumental learning paradigm that uses a medication as an unconditioned stimulus of which the physiological response is mimicked in response to a conditioned stimulus. This learning paradigm has later been applied to immune responses, in which an immune modulating medication is used as the physiological reaction, resulting in reduced immune medication dosages and maintained treatment efficacy in response to a stimulus (15). For instance, renal transplant patients, who received immunosuppressive treatment, were treated with a learned immunosuppressive placebo response, that was linked to a gustatory (conditioned) stimulus (70). When re-exposed to the conditioned stimulus, the T cell proliferative capacity was reduced in comparison to T cell functions under routine drug intake. Thus, classical pharmacological conditioning increased the medication efficacy.

## Behavioral factors associated to cancer onset

Numerous behavioral factors, of which stressors, exercise and conditioning are three examples, have been associated with the onset of cancer (71–74). According to the vulnerability-stress model, vulnerabilities (e.g. genetics) and stressors (e.g. life events) lead to certain behaviors (e.g. lifestyle) and physiological responses (e.g. immune responses) and can influence disease and clinical outcomes (36). Here, we focus on the above-mentioned behavioral factors that can be targeted in behavioral interventions and are exemplary of a spectrum of analogous behaviors. Long lasting stressors or a lack of physical exercise can substantially reduce tumor growth as shown in both epidemiological and in animal models, possibly mediated by immune cell modulation (34, 35). Classical pharmacological conditioning may be used to assist cancer treatment.

### Stressors

Confrontation with stressors can affect the immune system and increase cancer occurrence in human (75). The immune system mediates the relationship between stressors and cancer occurrence (76). Exposure to stressors is found to be accompanied by pro-inflammatory responses in animal and human research (77), which may stimulate tumor growth. Furthermore, stress related molecules, such as cortisol or the catecholamines (nor)epinephrine, can regulate diverse signaling pathways through their specific receptors that enhance the proliferative and invasive abilities of cancer cells in relation with the tumor microenvironment (78, 79). Cortisol or glucocorticoids have a pivotal role in regulating stress reactivity of organ systems (80) through glucocorticoid-receptor-mediated modulation of target genes (34). Glucocorticoids can activate survival genes that protect cancer cells from the effects of chemotherapy (81), and were shown to influence virus activation including human papillomaviruses and other cancer-associated viruses (34). Instead, to counteract the stimulating effects of (nor)epinephrine on tumor growth, the administration of beta blockers that interfere with the physiological stress response was associated with a lower incidence of prostate cancer in a population based study (82).

The influence of stressors on cancer incidence is also hypothesized to be moderated by socioeconomic status. Disparities in socioeconomic status are associated to inequalities in behavioral factors such as physical inactivity, obesity, smoking, diet, alcohol and drug use, screening and treatment uptake (83). These health-impairing behaviors are thought to be stress-related behaviors and a lower socioeconomic status has been associated with higher levels of distress (84). Higher cancer occurrence is found in groups with lower socioeconomic status (83, 85). Even though confrontation with long lasting stressors may be one of the factors that plays a role in these processes, research should detangle the mechanisms with which stress modulates cancer occurrence and therapy response.

### Exercise

In humans, pooled analyses of epidemiological studies showed that more physical activity during leisure time was associated with a decreased risk of 10 different type of cancers, independent of body mass index (BMI) (86). Overall, physical activity is associated with a 7%-20% lower cancer risk in individuals, with the strongest impact on colorectal and breast cancer (74, 87). One of the mechanisms by which physical activity may reduce risk of cancer occurrence is a reduction in chronic, low-grade inflammation and improved immune surveillance and function (88, 89). Cumulative evidence of both animal and humans studies shows that exercise modulates local and systemic inflammatory processes by altering both the number and function of circulating cells of the innate immune system (neutrophils, monocytes and NK cells), and of the adaptive immune system (T and B cells) (89). Exercise may also reduce the visceral fat mass, which is accompanied by less adipokine secretion and less macrophage infiltration into the adipose tissue, thereby reducing inflammation (90). Exercise also activates the HPA axis, initiating cortisol release, which in the case of exercise can contribute to an effective anti-inflammatory systemic host environment by downregulating cytokines as tumor necrosis factor (TNF)-α. The aforementioned physiological events activated by exercise are only examples among many other processes that have been detailed in a number of reviews (89–91). For example, exercise is accompanied by a higher level of catecholamines, such as (nor)epinephrine, which were related to similar anti-inflammatory effects as cortisol (90).

### Classical pharmacological conditioning

Different from psychosocial stress or exercise, which can also be part of lifestyle, classical pharmacological conditioning is always an intervention and, therefore, there are no epidemiological studies investigating this behavioral mechanism in relation to cancer onset.

## Behavioral factors enhance conventional cancer treatments

The behavior factors that we specified in our model have shown to influence conventional cancer treatments, such as chemotherapy, radiotherapy, and hormonal treatment. The immune system is one of the main links thought to connect behavioral factors to cancer therapy (92).

### Stress modulation in conventional cancer therapies

There are indications that pharmacological stress modulation can improve cancer progression. The physiological stress response seems to drive therapeutic resistance in murine tumor models (93, 94). The cellular and molecular microenvironment of cancer includes (peripheral) nerves that can modulate behavior or malignant cells, promoting tumor growth and illustrating the cross-talk between the neuroimmune system and cancer progression (35). Regulation of the tumor microenvironment by the sympathetic nervous system has been demonstrated in animal studies (95). Intratumoral neurotransmitters and neuropeptides have regulatory roles in the physiological and pathological functions of tissues, and emerging data suggest that cancer cells may take advantage of neurotransmitters-initiated signaling pathways to activate uncontrolled proliferation (96). For example, norepinephrine and epinephrine activate β-adrenoreceptors expressed on both cancer and immune cells thereby promoting growth of malignancies and inflammation. Moreover, these catecholamines can induce an endothelial cell metabolic switch mediated by β-adrenoreceptors resulting in increased tumor vascularization (96). The β-adrenergic pathway may be suppressed by beta blockers and as an example, it was shown that propranolol, a medication of the β-blocker class, was used to complement the treatment of several types of cancer, directly blocking cancer cell proliferation induced by epinephrine *in vitro* (97). This experimental evidence is supported by clinical studies that combine propranolol with other agents to stop metastasis (98), and epidemiological evidence showing that of 24,238 patients, the 12,119 propranolol users (for over six months) had lower risk of head and neck, esophagus, stomach, colon and prostate cancers (99). On the other hand, preclinical administration of dexamethasone, a synthetic glucocorticoid, induced chemotherapy and resistance in breast cancer, as well as *in vitro* tumor samples and cancer cell lines (100–102). This evidence underlines the importance of the HPA-pathway modulation in conventional cancer treatments.

While psychological interventions seem to influence immunity (103), evidence in relation with cancer remains very limited. Studies using psychological interventions in patients with cancer often did not assess treatment efficacy or health-related quality of life (104). The results of interventions on psychological wellbeing, such as cognitive behavioral therapy, in cancer treatment are variable finding mostly effects on outcomes such as anxiety, and fatigue (105). However, these interventions, e.g. cognitive behavioral therapy and mindfulness, cannot be tested in preclinical models.

### Exercise and conventional cancer therapies

There is both pre-clinical and clinical evidence for a relation between exercise and the immune system and effectiveness of exercise during chemotherapy or radiotherapy (16–26). In patients, exercise has been associated with reduced side effects of cancer and its treatment (27, 28). Thereby, exercise improved the physical and mental health and the overall self-reported quality of life of patients (27–29). Patient-reported outcome measures were complemented by immunological readouts, including the number of NK cells, expression of IL-6, or TNF-α production (16–26). In observational and randomized controlled exercise trials - both increases and decreases in defined immune markers were reported. For example, immune markers of NK cells differed: exercise had an inhibitory effect on the absolute number of NK cells in patients with breast cancer (106), an augmenting effect on NK cells percentages in patients with lung cancer (107), whereas no effect in another cohort of patients with breast cancer and other solid tumors was observed (17, 22). Comparably, IL-6 expression differed after exercise interventions, showing either a decrease in some studies (24–26), but no effects (17, 20, 23, 108), or an increase in other studies (16, 18, 109). Given the diverse effects that exercise has on the number of immune cells in patients with cancer, for instance in NK cells, it has been suggested that exercise may instead affect the cytotoxic activity of the immune cells, mirroring the effects exercise has on healthy individuals through inflammatory response pathways (110, 111). The large variation in the type of exercise interventions ranging from aerobic to resistance training may explain differences in exercise responses (26, 106, 107).

To date, most causal evidence of exercise on the anti-tumor efficacy of cancer treatment comes from animal studies (112, 113). A large advantage of these *in vivo* experiments is that there is little variation in interventions; i.e. most studies examined the effects of voluntary running. These experiments indicate that physical exercise modulates factors that are inherent to cancer treatment sensitivity, including the tumor microenvironment, e.g. hypoxia, tumor cell metabolism and tumor perfusion, next to having profound effects on immune cell populations (112–114). Illustrating these results, voluntary running tumor bearing mice experienced reduced tumor growth in diverse cancer models, e.g. lung cancer or myeloma, and displayed higher NK cell mobilization compared to sedentary control groups (113, 115, 116).

### Classical pharmacological conditioning of conventional cancer therapies

Conditioned effects in cancer patients during therapy were shown on outcomes such as nausea and immune modulation (117–120). Patients who were given a beverage prior to adjuvant chemotherapy experienced more nausea at later time points when they were confronted with the beverage alone compared to patients who did not receive a beverage before the therapy (118), and the other way around, e.g. a conditioning paradigm was applied to reduce nausea (120). Similarly, pediatric cancer patients undergoing chemotherapy showed increased natural killer cell activity and interferon-γ levels upon arrival at the hospital, when previously confronted with two cycles of chemotherapy in the hospital (117). These results may be seen as an indication that conditioned effects in cancer patients are possible on immune cell populations, however, the effects on cancer outcomes and therapy response remain to be investigated.

The mechanisms of how the immune system is conditioned are largely unknown. An association between the conditioned stimulus and the immune response needs to be established in the brain and conditioning thus relies on the interaction between the central nervous system and the immune system (15). Some murine studies demonstrated that lesioning of the insular cortex and central nucleus of the amygdala obstructs immunological conditioning, suggesting that these areas mediate conditioning (121, 122). Other murine studies hypothesize that continuous administration of substances, such as antigens, may be disruptive for the hosts’ homeostasis, and that conditioning may be a favorable alternative given a decreased substance administration (123). By linking the immune reaction with the central nervous system, it is assumed that the effect of the substance might be achieved without the disruptive effects of the substance (123). Furthermore, it is thought that different pharmacological conditioning paradigms rely on different mechanisms, given that the physiological reaction mimics diverse medication effects (124, 125). Despite little evidence on the pathways of this associative learning process, the conditioning paradigm has been successfully used in numerous rodent studies of nonconventional therapies, echoing the effects of cyclosporine A, opioids, lipopolysaccharide (LPS), lithium chloride, anti-lymphocyte serum, ovalbumin and bovine serum albumin using taste and odor as conditioned stimuli (126–132).

### Immunotherapy may be supported by behavioral factors

Immune suppressive mechanisms in cancer hamper effective immune responses (133). By therapeutically assisting anti-cancer immune responses, tumor growth and progression may be counteracted and cure can be promoted. Promising targets for immunological therapies are immune checkpoint proteins, which are used as a break in the immune system and consequently block over-activation of the immune system preventing autoimmunity (3). In addition, checkpoint signals are required for optimal T-cell recognition and generation of long-lasting T cell memory responses (3). One of the most well-known checkpoint proteins is Cytotoxic T lymphocyte antigen-4, or CTLA-4 (134), which is considered critical for maintenance of T cell homeostasis and tolerance (135). T cell activation requires engagement of the T cell antigen receptor-CD3 complex and ligation of costimulatory receptors, such as CD28, that bind to CD80 (B7.1) and CD86 (B7.2) on antigen-presenting cells. CTLA-4 is transported to the immunologic synapse when there is a potent or long-lasting stimulus (*via* the T cell receptor) (136), and outcompetes binding of CD28 to CD80 and CD86, hereby acting as negative regulator of proliferation and effector function of T cells (137). When tumor cells express ligands (e.g. CD80, CD86) for CTLA-4, T cell activity is inhibited after binding, hereby evading clearance by the immune system (138). Monoclonal antibodies, i.e. checkpoint inhibitors, can block CTLA-4, allowing activation of T cells and killing of tumor cells (7).

Programmed death (PD)-1 is another immune checkpoint molecule involved in regulating the balance between immune activation and tolerance, similar to CTLA-4 (134). Its ligands PD-L1 (B7-H1) and PD-L2 (B7-H2) are expressed on antigen presenting cells, but can be expressed on tumor cells as well, resulting in an immunosuppressive tumor microenvironment. Therefore, also anti-PD-1 or anti-PD-L1 monoclonal antibodies promote T cell-mediated tumor cell death (139, 140).

Immune checkpoint inhibitors (i.e. Ipilimumab (anti-CTLA-4), Nivolumab (anti-PD-1), Pembrolizumab (anti-PD-1), Atezolizumab (anti-PD-L1)) have been approved or are studied in clinical trials to treat multiple types of cancer of which melanoma and lung cancer respond best to therapy. Next to this, there are currently several antibodies and small molecules in development, targeting other immune checkpoints such as TIM3, CD39, B7H3, CD73, LAG3, and more (141–145).

Unfortunately, large groups of patients do not respond to or benefit from immunotherapy (8–10). It is not completely clear yet why some patients respond and others do not, which may have to do with tumor-intrinsic qualities. For instance, it was shown that high microsatellite instability (MSI) results into a high number of mutations and increased number of tumor-infiltrating lymphocytes (146, 147). As such, patients with an MSI-tumor are suitable candidates for immunotherapy. Nonetheless, even in tumors with high MSI observed response rates range between 30% and 50% (147), indicating that there are other factors that come into play besides the genetic and immunologic aspects of the tumor. Similarly, predictive biomarkers, like tumor-cell PD-L1 expression, are used to stratify the immunotherapy responders from the non-responders (148). However, PD-L1 testing alone is insufficient for patient selection in most malignancies and immune responses are not uniform across all malignancies. It is estimated that in the US 38% of patients with cancer are eligible for ICI therapy, given the molecular profile of their tumor, but only up to 11% respond to the ICI therapy (10). The remaining 27% of patients were eligible but did not respond, indicating the necessity for better predictive biomarkers, next to the need of increasing treatment sensitivity. Another drawback of checkpoint inhibition is that it is not cost effective in certain malignancies, with an economic benefit for choosing chemotherapy to treat i.e. recurrent or metastatic head and neck cancers and non-small cell lung cancers (12). Furthermore, both CTLA-4 and PD-1 blockade can have severe immune related autoimmune complications, for example side effects on the gastrointestinal tract, brain, thyroid, lungs and skin (3, 13, 14). In the light of the diverse drawbacks, efforts are needed to improve and support immunotherapy, enhance its anti-cancer effects and decrease the side effects.

**Figure 2:**
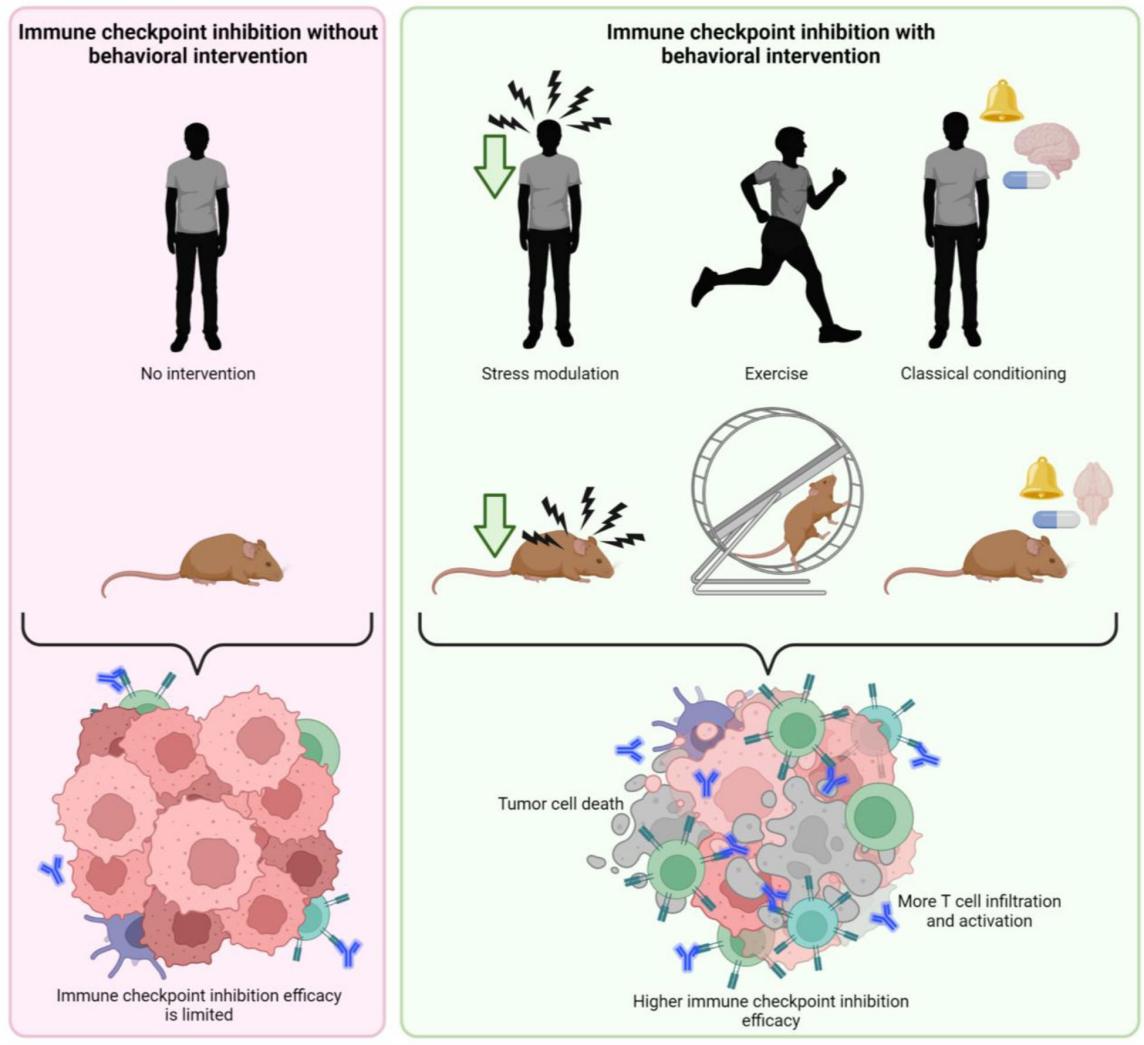
Putative effect of behavioral interventions on effectivity of immune checkpoint inhibition therapy. Behavioral interventions such as stress modulation, exercise and classical pharmacological conditioning may enhance the effectivity of immune checkpoint inhibition, promoting tumor cell death by higher T cell infiltration and activation created with BioRender.com.

Behavioral factors have been shown to influence both the immune system and cancer treatment and may therefore possibly offer opportunities to improve immunotherapy. Given that behavioral factors can influence the effectiveness of conventional chemo- and radiotherapy, and that the immune system is thought to modulate this effect, immunotherapy may offer an ideal opportunity for behavioral intervention (**Figure 2**).

### Physiological stress modulation and immune checkpoint inhibition

Few preliminary studies investigated the impact of stress modulation with pharmacological interventions on checkpoint inhibitors efficacyalthough there are indications that stress influences immunotherapy. For instance, social disruption stress compromised a vaccine based immunotherapy (poly(d,l-lactide-co-glycolide) microsphere) in a murine melanoma model through impairing CD8^+^ T cell responses (149). Also, either behavioral stress or surgical stress, weakened the inhibition of metastasis by immunostimulating agents (CpG class C and glucopyranosyl lipid-A stable emulsion) (150, 151). Studies linking stress to cancer immunotherapy assume the involvement of the of HPA-axis, for example by glucocorticoid-induced expression of the immunosuppressive transcription factor TSC22D3 of dendritic cells (152).

With regard to ICI in a retrospective analysis of 109 medical records of non-small lung cancer patients, treated with either ICI therapy or ICI in combination with chemotherapy, the 28 patients who were concomitantly prescribed any beta blocker had a longer progression free survival with a hazard ratio of 0.58 (153). A possible association between beta blocker use and improved progression free survival in non-small-cell lung cancer patients treated with ICI, should however be confirmed in clinical randomized controlled trials. In humans, there are no randomized controlled trials using psychological interventions to complement immune checkpoint inhibition cancer therapy.

An indication of a relation between stress exposure and the efficacy of anti-PD-L1 was found in a mouse study (154). In this study researchers applied daily chronic unpredictable mild stressors, such as water or food deprivation, tail pinching, bodily restraint et cetera, for a total of 28 days. The effect of anti-PD-L1 therapy was attenuated in the stressed groups, coinciding with a decrease in CD8^+^ lymphocytes and increase of regulatory T cells at tumor sites. In line with this, chronic cold stress strongly reduced anti-PD-1 efficacy in breast cancer and melanoma mouse models (155). An experimental group that experienced cold-induced stress (by being housed in an environment of 22 degrees Celsius instead of the thermoneutral temperature of 30 degrees Celsius) had larger tumors but this effect was counterbalanced by β-blocker treatment. The enhancement of anti-PD-1 efficacy by co-treatment with propranolol treatment was likely to be CD8^+^ dependent, i.e. there was an increased frequency of effector CD8^+^ T cells in subcutaneous breast cancer tumors and melanoma. The effects of propranolol treatment did not persist in T cell-deficient mice, suggesting that the β-adrenergic system influences T cell activity (155, 156). Similar results were found in mouse models of fibrosarcoma and colon cancers, where reduced tumor growth as well as enhanced response to anti-CTLA-4 therapy was observed after blocking the β-adrenergic receptor with propranolol (156). Here, propranolol treatment resulted in a reduction of tumor angiogenesis, increased T-cell infiltration, but a decrease in myeloid derived suppressor cells, as well as modifications on tumor associated macrophages, together leading to a tumor-suppressive environment (156). Despite these promising preliminary results, we are unaware of any ongoing clinical trials in humans.

### Exercise and immune checkpoint inhibition

Several murine studies investigated the synergistic effects of exercise and immune checkpoint inhibitors on cancer treatment (157–160). Higher tumor necrosis and less apoptosis was found in a patient-derived xenograft model of non-small cell lung carcinoma when anti-PD-1 treatment was combined with exercise, indicating that excise may improve anti-PD-1 effectivity (157). Another study demonstrated that aerobic exercise, i.e. daily 30 minutes treadmill exposure, sensitized pancreatic tumors to anti-PD-1 therapy, which resulted in anti-tumor immunity though IL-15Rα^+^ CD8^+^ T cells and decreased tumor growth (160). Two other *in vivo* studies found no synergistic effects of the combination of exercise and immune checkpoint inhibition. However, one study observed an increase in CD8^+^ T cells in orthotopically implanted breast tumors when anti-PD-1 was combined with voluntary wheel running, as compared to the tumors of mice without voluntary running regime (158, 159). These preliminary preclinical results offer a stepping-stone to translate exercise interventions to the clinic.

### Classical pharmacological conditioning and immune checkpoint inhibition

To the best of our knowledge there are no published studies investigating the effects of a learned immune reaction on the efficacy of immune checkpoint inhibition. However, a recent study showed that conditioning of rapamycin-induced immunomodulation reduced tumor growth effectively in a murine glioblastoma model (161). In this study, the mTOR inhibitor Rapamycin was repeatedly paired with a novel gustatory stimulus. The experimental group, receiving only 10% of the initial drug dose together with the gustatory stimulus during the testing phase, showed similar tumor inhibition as the control group receiving 100% of the drug dose. the tumor growth inhibition was driven by a central and peripheral upregulation of pro-inflammatory markers and a decrease in anti-inflammatory cytokines such as IL-10. Similarly, older studies showed that conditioning of immunotherapy was more effective in delaying tumor growth in mice than immunotherapy alone (162, 163). For example, in a syngeneic *in vivo* study, the unconditioned stimulus was the injection of immunostimulating DBA/2 spleen cells, and the conditioned stimulus was camphor odor. When conditioned mice were re-exposed to the odor of camphor only, tumor growth was still delayed compared to non-conditioned mice (162). These experiments suggest that the immune system of the mice consistently mimicked the effect of the immune modulator when presented with the conditioned stimulus, which influenced health outcomes, demonstrating the feasibility of conditioning immune responses (123, 161–167). Therefore, conditioned effects of immunomodulatory inhibitors may be suitable also for immune checkpoint inhibition. Of note, both mechanistic animal studies and controlled human studies in healthy subjects and patients are necessary to understand whether learned immunity is a promising addition to immunotherapy. In patients, the conditioning paradigm could be applied using specific stimuli, for instance combining the use of checkpoint inhibitors with a distinctive stimulus: a taste, sound or smell. This setting may serve for reducing medication dosages, i.e. checkpoint inhibition could be given in reduced quantities or placebo medication could be administered intermittently. Potentially, the use of mechanisms that harness mimicking placebo effects could reduce healthcare costs associated to the high expenses of several medications, including immune checkpoint inhibition.

Several ongoing clinical trials in patients study how behavioral factors may affect the quality of life and other cancer-related outcomes during ICI (**Table 1**). To the best of our knowledge there are no ongoing clinical trials on stress modulation or classical pharmacological conditioning in checkpoint inhibition, but various studies use exercise as intervention. The results of these studies are yet unknown and most studies focus on feasibility of the intervention as primary outcome measure. Therefore, the mechanisms of behavior on cancer outcomes in patients undergoing immune checkpoint inhibition therapy remain unknown.

**Table 1:** Ongoing studies researching behavioral factors and immune checkpoint inhibition therapy.

#	Study	Tumor	Intervention	N	Primary (1) and secondary (2) outcomes	Location	ID
1	Exercise to Boost Response to ICI	Cutaneous melanoma, cutaneous squamous cell carcinoma, Merkel cell carcinoma	30 minutes arm ergometer/pedal ergometer/treadmill exercise up to 12 times and 12 months prior to each administration of standard of care checkpoint blockade immunotherapy across all cycles	32	1: Feasibility of the exercise intervention 2: Tumor Immunological Biomarkers	Moffitt Cancer Center Tampa, Florida, USA	NCT05358938
2	Exercise as a Supportive Measure for Patients Undergoing ICI	Melanoma	60 minutes group, machine based resistance and endurance exercise (moderate-to-high-intensity), 2 times a week for 12 weeks	40	1: Feasibility of the exercise intervention 2: Quality of life (EORTC QLQ-C30, version 3.0), fatigue (MFI), sleep Quality (PSQI), depression (CES-D), physical Activity (SQUASH), cardiopulmonary fitness (maximal aerobic capacity (VO_2_peak) *via* a maximal incremental cycling test), muscle strength (isometric and isokinetic with the Isomed 2000^®^ diagnostic module), pain (BPI)	Heidelberg University Clinic, Heidelberg, DE	NCT03171064
3	Low-moderate Intensity Pedaling During Immunotherapy Administration	Skin, kidney, bladder cancer	30 minutes on a pedal ergometer (low-moderate intensity) concurrent to ICI infusion for maximum 12 weeks	10	1: Feasibility of pedaling measured by the number of completed pedaling sessions and the ability of patients to meet pedaling intensity goals. 2: Quality of life scores (Quality of Life Questionnaire - Core 30), treatment response biomarkers (checkpoint inhibitors, functional T and B cell subsets, pro and anti-inflammatory monocyte subsets, and soluble inflammatory mediators), CT-derived sarcopenia rates	Rush University Medical Center, Chicago, Illinois, USA	NCT04127318
4	Combined Aerobic and Resistance Exercise Training in Metastatic Renal Cell Carcinoma	Renal cell carcinoma	12 weeks home based, combined aerobic and resistance exercise training plan	16	1: Feasibility of the exercise intervention 2: Change in Health Related Quality of Life (FACT-G), the incidence of grade 3-5 toxicities as per CTCAE 5.0	Johns Hopkins, University/Sidney Kimmel Cancer Center, Baltimore, Maryland, USA	NCT05103722
5	i-Move	Melanoma	12-week semi-supervised individualized exercises: moderate intensity aerobic exercise (walking and cycling, 20-45 min, 3-5 times a week), resistance training exercises (2-3 times a week) and stretching	30	1: Feasibility of the exercise intervention 2: Fatigue (FACIT F), functioning (PROMIS), symptoms (Edmonton Symptom Assessment Scale), quality of life (SF-36), adherence (Godin Leisure-time Physical Activity Questionnaire), physical fitness and functioning (30 s chair stand test, the 6 min walk test, the arm curl test and the Australia-modified Karnofsky Performance Scale)	Peter MacCallum Cancer Centre, Melbourne, Australia	ACTRN 12619000952145

EORTC QLQ-C30, European Organization for the Research and Treatment of Cancer Quality of Life Questionnaire C30; MFI, Multidimensional Fatigue Inventory; PSQI, Pittsburgh Sleep Quality Index; CES-D, Center for Epidemiological Studies Depression Scale; SQUASH, Short QUestionnaire to ASsess Health-enhancing Physical Activity; BPI, Brief Pain Inventory; FACT-G, Functional Assessment of Cancer Therapy – General; CTCAE 5.0, Common Terminology Criteria for Adverse Events 5.0; FACIT F, Functional assessment of chronic illness therapy-fatigue; PROMIS, Patient-Reported Outcomes Measurement Information System V2.0; SF-36, Short Form 36 Health Survey Questionnaire.

## Conclusion

**Figure 3:**
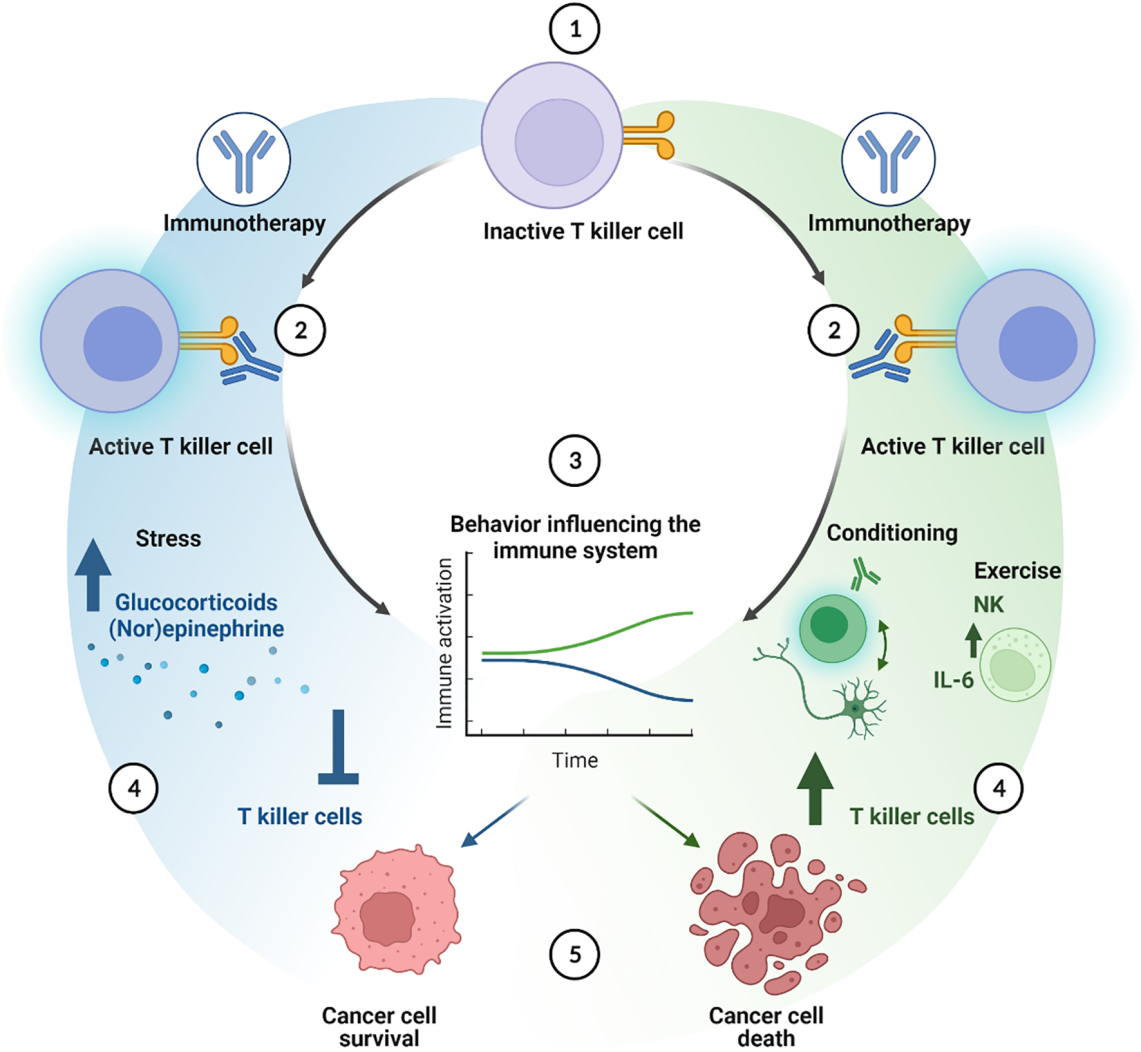
Cellular cascade of additive effects of behavioral interventions on immune checkpoint inhibition therapy. Immune checkpoint inhibition activates T killer cells (1 and 2). Behavioral factors or interventions can either improve (green line) or decrease (blue line) the effects of the immune therapy (3). Stress hampers the immune system through for instance the effects of glucocorticoids and (nor)epinephrine, inhibiting T killer cell function and numbers, thereby enhancing cancer cell survival (4 and 5, blue cascade). Classical pharmacological conditioning can enhance T killer cell activity and numbers mediated by brain-immune communication. Exercise may increase the amount of NK cells, induce IL-6 secretion, altogether improving T killer cell activation, leading to a better anti-tumor response (4 and 5, green cascade) created with BioRender.com.

Behavioral factors are associated with the onset and therapy response of cancer. Behavioral interventions such as modulation of psychosocial stress, exercise, and classical pharmacological conditioning, have therefore been used to reduce toxicity and potentially improve conventional cancer therapy outcomes. In **Figure 3**, we summarize how behavioral factors might affect the individual immune cell types. With the rise of various immunotherapies that counteract the immune suppressing interactions between tumors and the immune system, there are ample opportunities for non-invasive behavioral interventions to improve immunotherapeutic results. Hence, it is of paramount importance to rigorously examine the potential advantageous effects of behaviors that may support tumor cell clearance by the immune system activated by ICI therapy. The efficacy of these behavioral factors remains to be tested both in animal models, to investigate underlying mechanisms, and in patients, to explore their suitability for the benefit of cancer therapy.

## Data availability statement

The original contributions presented in the study are included in the article/supplementary material. Further inquiries can be directed to the corresponding author.

## Author contributions

All authors have made substantial contributions to the conception or design of the work; have drafted and revised the work; and have provided approval for the publication of the content.
